# Survival of Polyploid hybrid salamander embryos

**DOI:** 10.1186/s12861-019-0202-z

**Published:** 2019-11-12

**Authors:** Noah D. Charney, Jacob E. Kubel, Craig T. Woodard, Blanca I. Carbajal-González, Samantha Avis, Julia A. Blyth, Charles S. Eiseman, John Castorino, John H. Malone

**Affiliations:** 1 Harvard Forest, Harvard University, Petersham, MA USA; 2 Natural Heritage & Endangered Species Program, Massachusetts Division of Fisheries and Wildlife, Westborough, MA USA; 3 Mount Holyoke College, South Hadley, MA USA; 4 Maria Mitchell Association, Nantucket, MA USA; 5 Northfield, MA USA; 6 Hampshire College, Amherst, MA USA; 7 Department of Molecular and Cell Biology, University of Connecticut, Storrs, USA

**Keywords:** Ambystoma, Development, Fertilization, Gene regulation, Gynogenesis, Kleptogenesis, Maternal-to-zygotic transition, Mortality, Oogenesis, TUNEL

## Abstract

**Background:**

Animals with polyploid, hybrid nuclei offer a challenge for models of gene expression and regulation during embryogenesis. To understand how such organisms proceed through development, we examined the timing and prevalence of mortality among embryos of unisexual salamanders in the genus *Ambystoma.*

**Results:**

Our regional field surveys suggested that heightened rates of embryo mortality among unisexual salamanders begin in the earliest stages of embryogenesis. Although we expected elevated mortality after zygotic genome activation in the blastula stage, this is not what we found among embryos which we reared in the laboratory. Once embryos entered the first cleavage stage, we found no difference in mortality rates between unisexual salamanders and their bisexual hosts. Our results are consistent with previous studies showing high rates of unisexual mortality, but counter to reports that heightened embryo mortality continues throughout embryo development.

**Conclusions:**

Possible causes of embryonic mortality in early embryogenesis suggested by our results include abnormal maternal loading of RNA during meiosis and barriers to insemination. The surprising survival rates of embryos post-cleavage invites further study of how genes are regulated during development in such polyploid hybrid organisms.

## Background

The typical diploid eukaryote has two alleles for each autosomal locus that are expressed at levels necessary for proper development and genome function. The dynamics of this two-allele system are especially important for transcription factors that bind to *cis* regulatory regions and protein macromolecular complexes, in which proper stoichiometry is needed for binding and for proper complex assembly. The impact of abnormal dosage and reaction kinetics remain difficult to study because divergent gene sequences and abnormal copy number are normally deleterious [10]. Organisms with polyploid genomes derived from highly divergent species challenge traditional models of *cis* and *trans* regulation and present novel systems for studying the impact of these factors on early development [11]. Understanding how polyploid organisms successfully complete oogenesis, fertilization, and embryonic development should provide valuable insights into the impacts of abnormal chromosome/gene dosage and regulatory reaction kinetics on embryonic development [65].

An exemplary system for understanding the challenges of polyploid reproduction is the unisexual salamander complex of eastern North America. This complex consists of five bisexual host species (*A. barbouri*, *A. jeffersonianum*, *A. laterale*, *A. texanum*, *A. tigrinum*) and a suite of unisexual associates of varying ploidy (diploid to pentaploid) and genome composition [14, 20, 21]. In our study region of New England, the host species are *A. jeffersonianum* and *A. laterale.* Regardless of context, the mitochondria of all unisexuals can be traced back to a single origin. Despite the theoretical challenges of hybrid polyploid genomes, and despite evolutionary expectations that unisexual vertebrates should be short-lived, this lineage has been thriving for five million years [6].

Reproduction among unisexual salamanders differs from other known modes of biological reproduction, which include: normal sexual reproduction, where males and females contribute equal amounts of nuclear DNA to offspring; parthenogenesis, where females clonally reproduce in the absence of males; gynogenesis, where paternal sperm is used only to activate embryo development and none of the paternal nuclear DNA is incorporated into the offspring; and hybridogenesis, where the paternal nuclear DNA is expressed in the offspring, but is not transmitted to subsequent generations [50]. Unisexual salamander reproduction often takes a gynogenetic pathway, where sperm is used only to stimulate embryo development, essentially resulting in cloning of the unisexual ([28, 32, 46]; but see [7]). However, some of the time, sperm nuclei are incorporated, elevating the ploidy of offspring and transmitting whole sets of paternal genomes to future generations, similar to normal sexual reproduction [15, 20, 32, 56, 60].

Although unisexual salamanders typically dominate populations where they co-occur with the bisexual salamander hosts [24], very high rates of embryo mortality occur in populations where unisexuals are present and this phenomenon is well-documented throughout their range [20, 26, 41, 49, 53, 66]. Within these ponds, some researchers [19, 43, 55] have reported that the rates of mortality are higher among unisexual salamanders than their bisexual hosts, while others have not [18, 40]. There is some evidence that tetraploid and pentaploid unisexuals are more prone to developmental defects and larval mortality than are triploids [45, 55, 63]. High rates of embryonic mortality suggest that there are substantial costs associated with this life history strategy, and that these costs arise in the course of embryogenesis – during oogenesis, fertilization, or embryonic development.

During egg development, genomes of unisexual organisms such as the unisexual salamanders are thought to undergo a doubling event, known as pre-meiotic endomitosis [5, 46]. Because these nuclei begin with polyploid genomes, this meiotic pathway entails stages with very high numbers of genome copies. For example, in a tetraploid (*n* = 4) unisexual, after the doubling phase associated with meiosis, the nuclei may contain 16 homologous chromosome sets prior to division. Meiosis is further complicated by the fact that unisexual salamanders carry genome sets that are hybrid combinations of other species’ distinct genomes. Due to these complexities, unisexual meiosis is prone to errors, such as improper chromosome segregation [3, 22], intergenomic recombination, and translocations, all of which can lead to embryonic mortality ([4, 7–9, 20].

When amphibian eggs are inseminated, the sperm induces an increase in intracellular Ca^2+^ concentration in the egg, which is both necessary and sufficient for egg activation [35]. To activate development of their eggs, unisexual *Ambystoma* females use sperm from males of normally bisexual species [20]. When reproduction follows a gynogenetic pathway, the sperm nucleus is then discarded, as observed in most of the samples examined by Elinson et al. [32]. During the first cleavage following fertilization, Elinson et al. [32] observed an uncondensed clump of chromatin which they interpreted as the sperm nucleus. This clump remained at the metaphase plate – which subsequently became the site of furrow formation – while the other chromosomes retreated to separate nuclei.

The early stages of embryonic development rely on RNAs deposited into the egg by the mother during oogenesis [62]. During the maternal-to-zygotic transition (MZT), transcription of genes in the zygotic nucleus begins, and expression of maternal transcripts declines. The successful MZT is essential for embryonic development and requires large-scale zygotic genome activation (ZGA), which occurs during the mid-blastula transition (MBT [39, 47, 62];). Findings from studies with amphibian and fish embryos suggest that there is a developmental checkpoint activated at the MBT that eliminates abnormal cells by apoptosis [1, 31, 37, 61]. Several different, but not mutually exclusive models for the mechanisms underlying the timing of ZGA have been proposed [29, 36, 37, 39, 51, 52, 68]. Early embryogenesis, and each of the hypothesized mechanisms for ZGA timing could potentially be compromised in embryos with multispecies hybrid polyploid genomes, possibly resulting in developmental abnormalities, death of cells by apoptosis, and embryonic mortality.

In addition to the aforementioned challenges for unisexuals in meiosis and ZGA, proposed explanations for observed embryonic mortality in the unisexual salamander complex have included low water temperature, polyspermy, “over ripeness of eggs” resulting from delayed reproduction, and poor rates of egg fertilization or activation [26, 40, 54]. Because different mechanisms are engaged in successive stages of development, understanding the timing at which embryos die will go a long way towards a full understanding of how these polyploid organisms ultimately persist despite the theoretical challenges.

In this study, we investigated the timing and prevalence of unisexual embryo mortality. Field biologists typically associate the presence of high rates of visibly dead embryos as a characteristic feature of ponds where unisexual salamanders are present. By contrast, in ponds where unisexual salamanders are not present and in egg masses of other *Ambystoma* species, such high mortality rates are only occasionally observed, and may be attributed to environmental stressors such as freezing or road salt in ponds. Given our understanding that genetic errors in the developing embryos contribute to unisexual embryo mortality, we expected that elevated rates of unisexual salamander embryo mortality will begin during or after the blastula stage, when zygotic genes take over control of development, with high rates of mortality continuing through subsequent developmental stages.

To understand mortality during unisexual development, we examined three different data sets, which offered tradeoffs between precision and generalizability. To characterize embryo mortality associated with the unisexual salamander complex at the broadest level, we analyzed mortality in field photos of egg masses from 137 ponds. Based on previous work [24], it is assumed that the majority of these eggs were from unisexual salamanders. These data allow inference to regional patterns of mortality, providing a link between our more-detailed laboratory studies and the observations of widespread embryo mortality typically reported by field biologists. However, without genetic data, we cannot use this broad data set to discriminate mortality rates between unisexual and *A. jeffersonianum*. From a set of 10 ponds, we also collected genetic data allowing us to discriminate mortality between the two maternal lineages. For the most granular perspective on the timing of mortality from the cleavage stage forward, we tracked mortality in 11 developing egg masses collected from 3 ponds and reared in the laboratory.

## Results

### Field observations – maternal lineage known

**Fig. 1:**
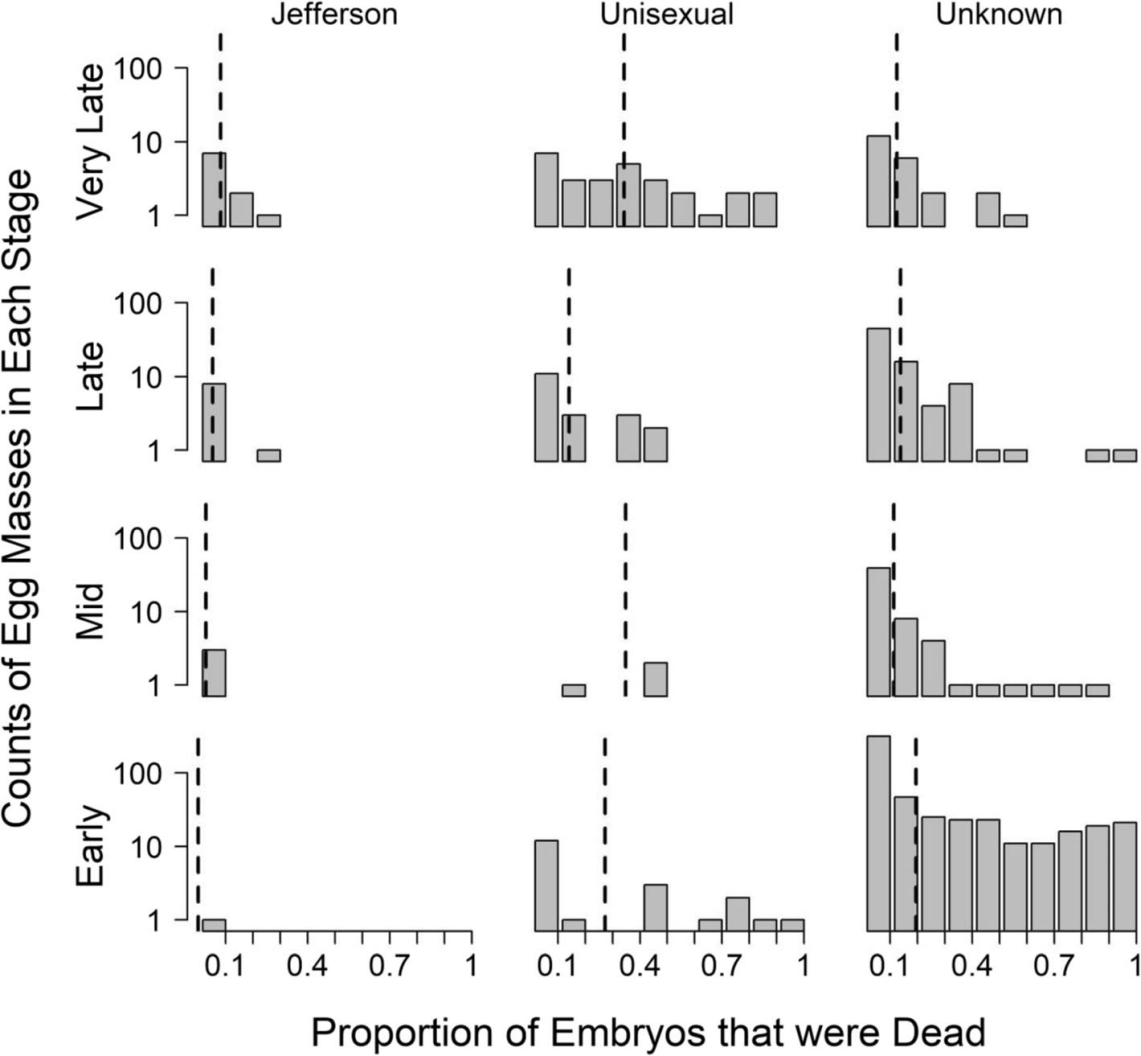
Histograms of the proportion of embryos that were dead in field photographs of egg masses with genetically determined maternal lineages (Jefferson or Unisexual) and egg masses from the same complex of unknown maternal lineage. The dotted vertical line in each group represents the arithmetic mean. The y-axis represents counts in each bin, and is arranged along a log scale

**Fig. 2:**
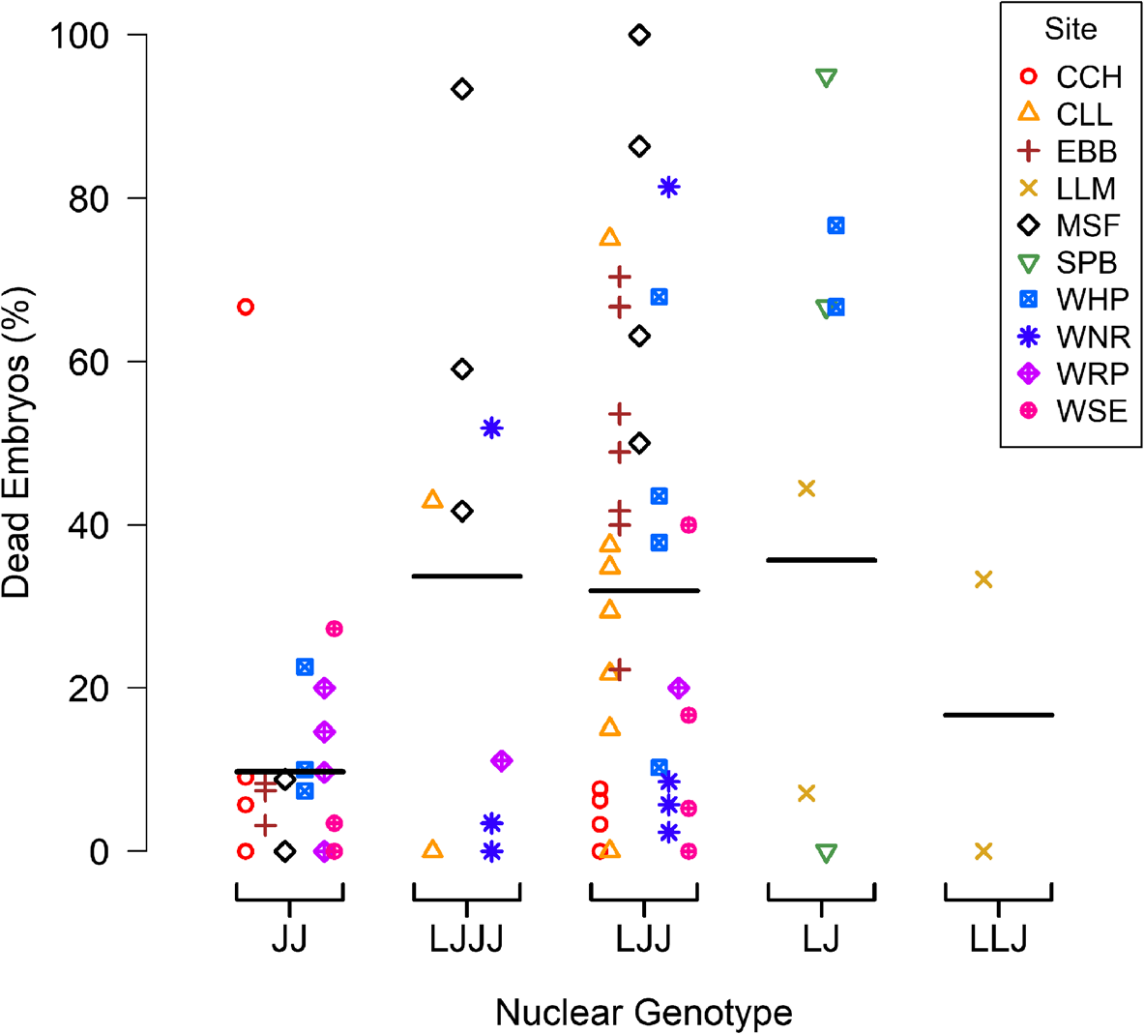
Observed frequency of dead embryos in field surveys at 10 sites across Massachusetts. Each point represents a whole egg mass, with genetic identity inferred by microsatellite markers from a single embryo taken from the egg mass, whereas nuclear genotypes are known to vary amongst embryos in a mass. The horizontal lines represent the mean percent dead embryos among masses within each genotype group. In describing nuclear genotypes, the number of times each letter is repeated represents the number of copies of each genome set, with “L” for *Ambystoma laterale* and “J” for *A. jeffersonianum*

To characterize the phenomenon of elevated embryo mortality in populations where unisexual salamanders occur, we observed 94 egg masses with genetically identified maternal lineages in the field during 2017. These consisted of 23 *A. jeffersonianum* and 71 unisexual masses, with 56 and 15 of the unisexual masses associated with populations of *A. jeffersonianum* and *A. laterale*, respectively [25]. Despite including ponds where *A. laterale* was the host, we did not observe any *A. laterale* eggs, largely because the eggs are typically laid singly and difficult to detect [25]. The percent dead embryos were significantly greater (*p* < 0.001) in unisexual (mean = 29%, sd = 32%) than in *A. jeffersonianum* (mean = 10%, sd = 16%) masses, even after controlling for site and developmental stage (Fig. 1). The average observed developmental stages were slightly earlier in unisexual (mean = 26, sd = 14) than *A. jeffersonianum* (mean = 32, sd = 6), but this difference was not significant after controlling for site (*p* = 0.06). The unisexual salamanders in our study included four different nuclear genome combinations, LJJJ, LJJ, LJ, and LLJ, specified by the number of copies of *A. laterale* (L), and *A. jeffersonianum* (J) genome sets. Among unisexuals, the rates of mortality were similar across nuclear genome combinations (Fig. 2). High rates of mortality were observed beginning early in development (defined here as the stages up through the beginning of neurula): for 23% (5 out of 22) of the unisexual egg masses in early developmental stages, we observed that more than half of the embryos in each mass had already turned white or were showing other outward signs of decay. Early-stage unisexual egg masses had on average 27% (sd = 35%) dead embryos.

### Field observations – maternal lineage unknown

For a broader-scale characterization of embryo mortality in unisexual populations, we examined field photos collected from 137 ponds during regional surveys. After excluding 109 images that were of low quality, we counted embryos in a total of 663 distinct sets, representing unknown ratios of Jefferson and unisexual salamanders. Overall, the mean percent of dead eggs in these egg masses was 18% (sd = 27%). Among early-stage egg masses, 15% (78 out of 506) already displayed mortality in more than half of the embryos in each mass. The average mortality across early stage embryos was 19% (sd = 29%).

### Laboratory reared specimens

**Fig. 3:**
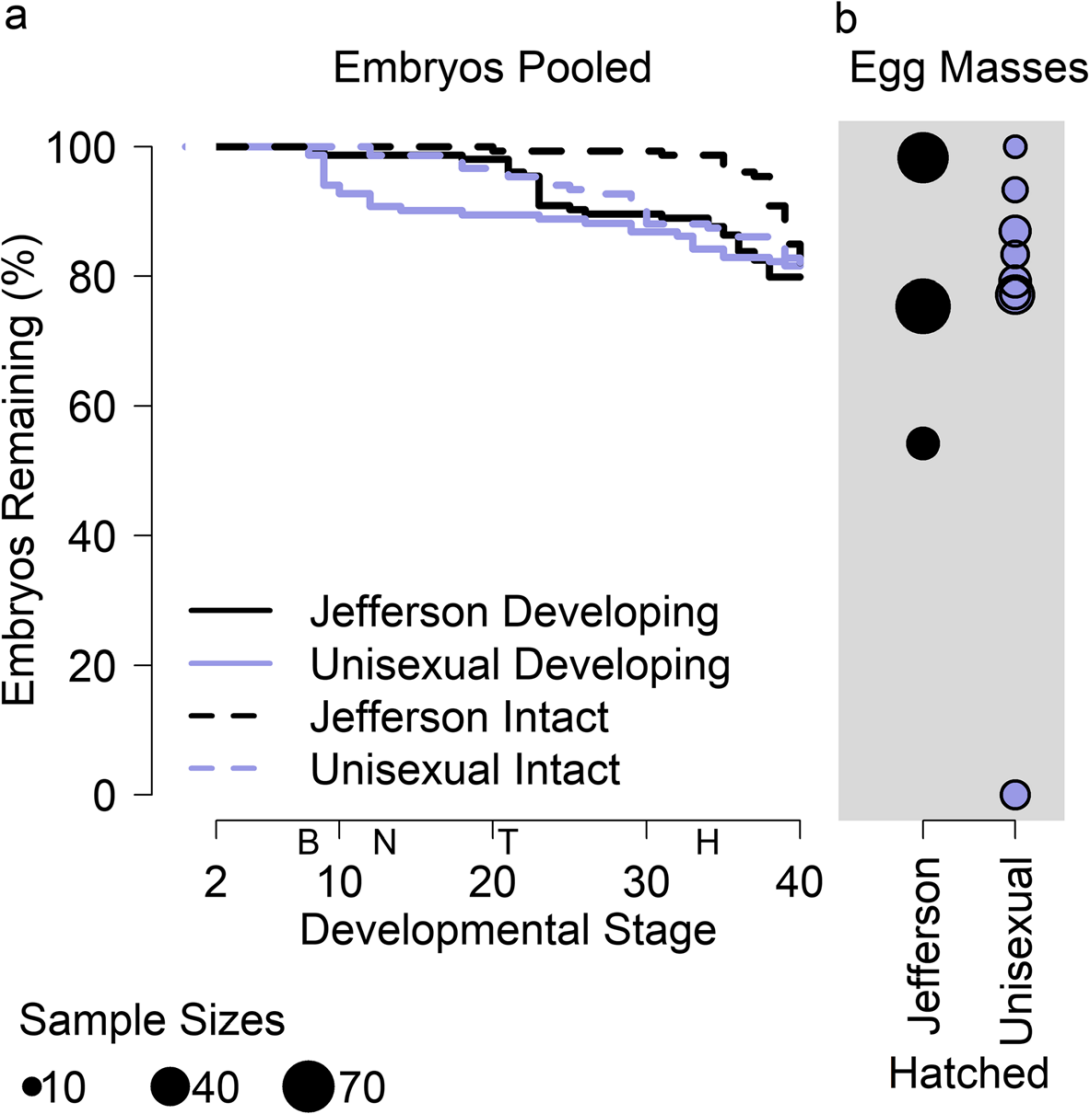
Survival of lab-reared Jefferson and unisexual salamander embryos, excluding those sacrificed for further laboratory testing. **a** Survival curves of all embryos that entered cleavage (stage 2). Solid lines track survival until development stopped advancing. Dotted lines track survival until embryos turned white across expected developmental stage, which is based on the stage of neighboring embryos in the egg mass when the focal embryo turned white. The horizontal space between solid and dotted lines represents the approximate delay between embryo death and the outward appearance of decay. The letters on the x-axis represent the blastula stage (“B”), the beginning of the neurulation (“N”), the beginning of the tail-bud (“T”), and the beginning of the heartbeat (“H”). **b** Hatching success, where each point represents one egg mass. The area of the circles reflect the relative number of embryos (ranging from 12 to 69) in each mass. None of the embryos in the one egg mass with complete mortality entered the cleavage stage, and so this egg mass is not represented in the survival curves to the left. All embryos in all other masses entered cleavage

For a fine-grained look at embryo mortality after development is initiated, we tracked 321 developing eggs in 11 masses in the laboratory, consisting of 152 eggs in 3 *A. jeffersonianum* masses (B, G, and H; Table 1) and 169 eggs in 8 unisexual masses (A, C–F, and I–K). The mean mortality rate among the 11 masses was 25% (sd = 28%), with no significant difference (*p* = 0.4) in mortality rate between *A. jeffersonianum* (mean = 24%, sd = 22%) and unisexual (mean = 25%, sd = 31%) embryos (Table 1, Fig. 3). Pooling the embryos across all egg masses from each maternal lineage, and excluding both embryos sampled for genetic analysis and those not entering the first cleavage stage, we observed mortality in 19% (29 out of 152) and 17% (25 out of 149) of developing *A. jeffersonianum* and unisexual embryos, respectively (Table 1).

**Table 1:** Hatching outcome of embryos among 11 salamander egg masses collected from western Massachusetts during early stages of development and tracked in the lab, 2016

Lineage	Town	Mass ID	Initial Stage	Embryo Count^a^	Hatch Count	Hatch Fraction
Jefferson	Sunderland	B	4	69	52	0.75
Jefferson	W. Springfield	G	1	59	58	0.98
Jefferson	Lenox	H	2	24	13	0.54
	Jefferson Subtotal			152	123	0.81
Unisexual	Sunderland	A	6	35	27	0.77
Unisexual	W. Springfield	C	8	15	14	0.93
Unisexual	W. Springfield	D	6	23	20	0.87
Unisexual	W. Springfield	E	1	24	19	0.79
Unisexual	W. Springfield	F^b^	1	20	0	0
Unisexual	Lenox	I	2	18	15	0.83
Unisexual	Lenox	J	3	12	12	1
Unisexual	Lenox	K	3	22	17	0.77
	Unisexual Subtotal			169	124	0.73

^a^Counts do not include embryos excised for genetic analysis

^b^No embryos in mass F entered first cleavage stage

**Fig. 4:**
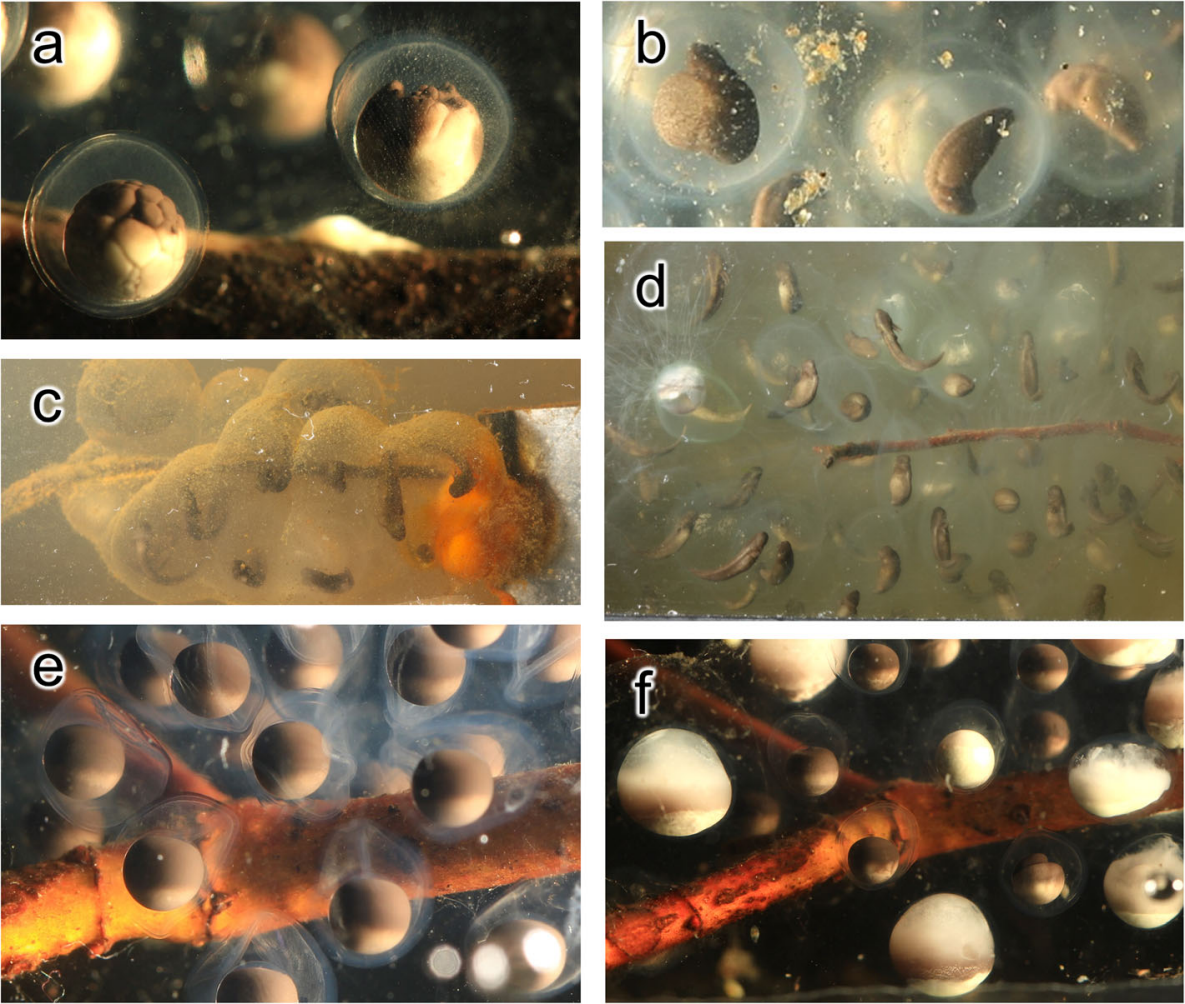
Differing pathways of embryo failure. **a** Blebbing and mold filaments on right embryo of unisexual mass E, **b** ballooning abdomen in left embryo of unisexual mass A, **c** bacterial bloom on unisexual mass K, **d** aborted development of many embryos of Jefferson mass B, **e** non-spherical membranes in non-developing embryos of unisexual mass F, and **f** varying stages of decay among these same embryos in mass F

The patterns of embryo death differed between egg masses, with causes that could varyingly be attributed to pre-cleavage factors, environmental factors, and genetic factors (Fig. 4; Supplemental Results). In embryos that died, development terminated at various stages, ranging from early to late development (Fig. 3). Once development was terminated, the embryos typically did not exhibit outward signs of decay until the other embryos in the mass had advanced through later stages of development. Only unisexual mass J experienced no mortality, with all 12 embryos hatching successfully. All embryos in all egg masses entered cleavage, except for the embryos in unisexual mass F, which suffered complete embryo mortality with none of the eggs entering the first stage of division (see animation supplement archived on Dryad, 10.5061/dryad.rxwdbrv40).

**Fig. 5:**
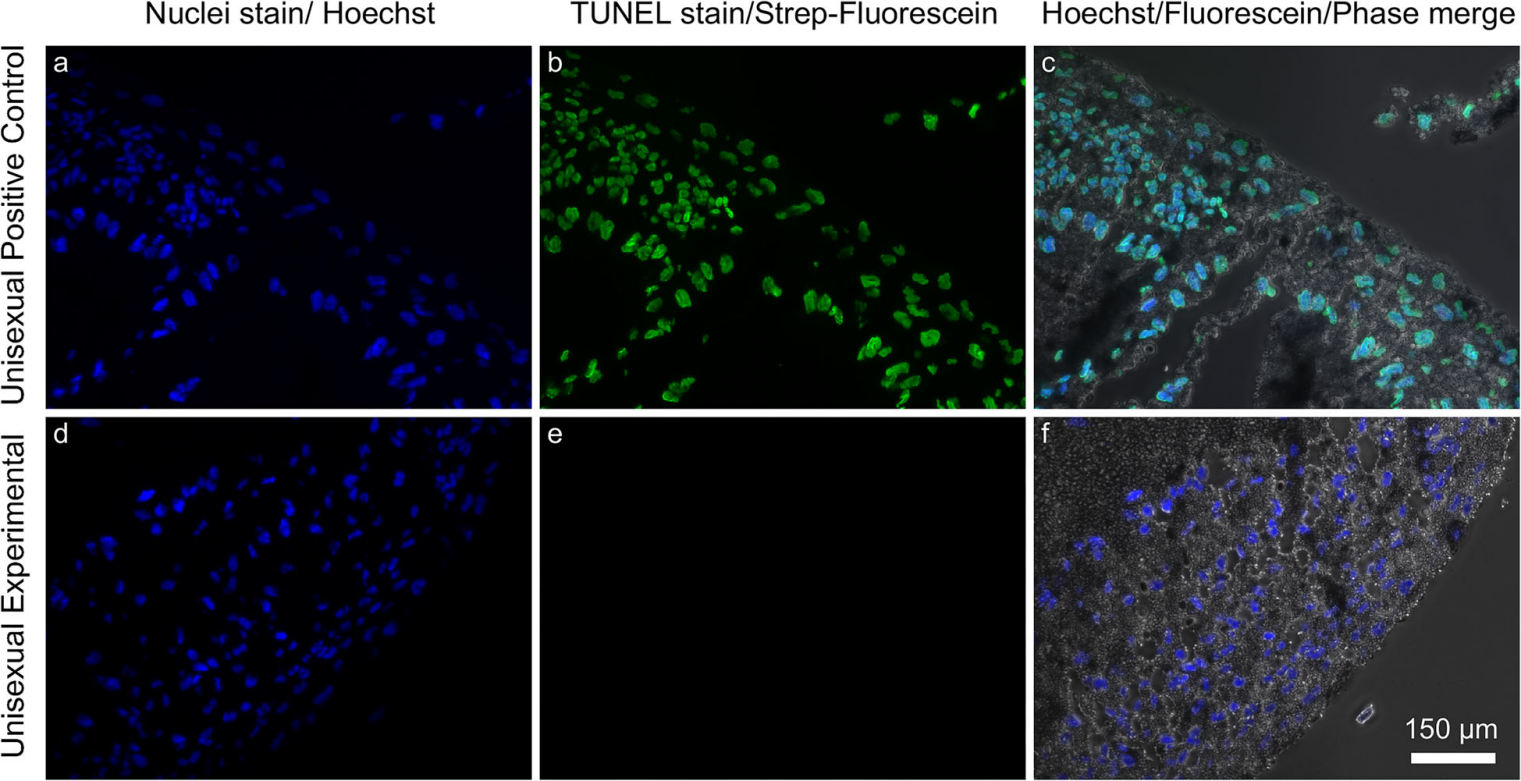
Microscopic analysis and apoptosis assay in a representative Stage 22 unisexual *Ambystoma* embryo. **a** and **d** Hoechst stain (blue fluorescence) indicates locations of cell nuclei. **b** Unisexual positive control, treated with TACS-NucleaseTM (Trevigen), a proprietary endonuclease, exhibits positive TUNEL staining as is evident by green fluorescence indicating the presence of DNA damage. **e** Experimental unisexual sample exhibits negative TUNEL staining (no green fluorescence), indicating absence of DNA damage characteristic of apoptosis. **c** and **f** Hoechst stain and TUNEL labeling merged with phase contrast microscopy

While most of our study is based on observations of embryo death, abnormal development may not be immediately lethal to embryos. Abnormalities in processes such as chromosome replication and ZGA may induce apoptosis or necrotic death at the cellular level early in development, although death of the entire organism may be delayed until a later stage of embryo development or until after hatching. To examine cellular-level developmental abnormalities, we fixed, sectioned and imaged a subset of embryos using phase contrast and fluorescence microscopy. We also used the TUNEL assay to detect apoptotic cell death. Comparison of the unisexual embryos with the Jefferson embryos revealed no detectable major differences in morphology (Fig. 5). The tissues did suffer some mechanical damage during our experimental procedures, so it is impossible to say that they were all completely normal. We saw no TUNEL-positive staining in any of the developing Jefferson or unisexual embryos we examined, indicating that the embryos were not undergoing aberrant cell death at the developmental stages assayed (stages 8+ through 25) (Fig. 5). The positive control, in which nuclear DNA is cleaved artificially with TACS-Nuclease™ (Trevigen), a proprietary endonuclease, had strong TUNEL-positive FITC signal that corresponds to the location of nuclei.

## Discussion

Many previous studies have described elevated rates of egg mortality in populations where unisexual salamanders are present, but there is still little understanding of the underlying mechanisms. Our findings suggest that, within our region of the unisexual complex, the characteristic elevated embryo mortality occurs in the earliest stages of embryogenesis. This is counter to our expectations that high mortality would set in after the blastula stage, but is supported by all aspects of our study.

In the field, we observed high rates of mortality among early stage embryos. In the controlled laboratory experiment, once the embryos entered cleavage, the unisexual and *A. jeffersonianum* embryos survived to hatching at approximately the same rate, and in our microscopic and TUNEL staining analyses we saw no signs of gross developmental abnormalities or cell death among developing embryos. The only catastrophic failure observed in the lab was in unisexual mass F, in which no embryos entered cleavage.

Previous studies showing high mortality among laboratory-reared unisexual embryos did not offer controlled side-by-side comparisons with bisexual hosts (e.g. [19, 32]). For instance, Elinson et al. [32] found that “only 75% of cleaved eggs developed to hatching,” and they later suggest that genetic factors may be one cause for low survival rates among unisexuals. Our survival rate post-cleavage in the laboratory was similar (83%), however we do not interpret this as low, because we saw similar survival rates of *A. jeffersonianum* reared in the same experiment (81%). Elinson et al. [32] also found that only 50% of embryos successfully cleaved, which is consistent with our interpretation that the bulk of mortality among unisexual eggs occurs prior to cleavage.

That we don’t see higher levels of mortality once development begins is surprising. The unisexual salamanders in our region are primarily triploid, with nuclei that are hybrid combinations of two different host species (*A. laterale* and *A. jeffersonianum;* the dominant nuclear genotype in our study was LJJ), and mitochondria that are more closely related to a third species (*A. barbouri*) than either of the two host species [57].

Although the unisexual mitochondria form a 5-million year old monophyletic lineage, there has been continual turnover in nuclear genes over this time, with one-directional flow of genes from the local host species to the unisexuals [23]. Because the host species do not receive genes from unisexuals and genomic combinations arise de-novo based on local host species, natural selection has had limited opportunity to eliminate incompatibilities within the combinations of nuclear genomes carried by unisexuals. The two genomes that co-occur in unisexuals are widely divergent and, therefore, numerous between-species genetic incompatibilities should be present. The extent of these incompatibilities normally would lead to changes in gene regulation and produce abnormal development after MBT, as well as nuclear/mitochondrial incompatibilities that would affect cellular respiration. Indeed, in other parts of the unisexual range, authors have reported very low survival, with hatching rates around 20% [19, 20]. However, the salamanders in the regions covered by those studies have nuclear genomes that combine even more divergent species than *A. jeffersonianum* and *A. laterale*, which are more closely related to each other than to any other member of the unisexual complex. In other regions of the unisexual complex, *A. laterale* genomes can be found in a myriad of hybrid combinations with the more distantly related *A. tigrinum, A. texanum,* and *A. barbouri* nuclear genomes [21, 67], and so higher rates of mortality may be expected.

Death in early embryogenesis suggests a few possible mechanisms. Recent literature suggests that embryo mortality may be linked to genetic errors stemming from meiosis [20]. Unisexual reproduction is complex, and involves very large numbers of copies of genome sets in the lead up to meiosis [5, 6]. This can result in aneuploidy or other alignment failures [8], as well as errors due to recombinations between *A. jeffersonianum* and *A. laterale* genomes which constitute their nuclei [4, 7].

However, if genetic errors in the nucleus are the cause of mortality and mortality occurs prior to cleavage, this presents a potential paradox given our understanding of development. In animals, the first few stages of development are controlled by the maternal genome via cytoplasmic elements. The zygotic genome does not begin to exert control on development until the initiation of the major ZGA [39, 47, 62, 68]. This occurs during the blastula stage, which corresponds approximately to stage 8 in the numbering system we use here [58]. This raises the question as to how genetic errors within the zygote nucleus stemming from meiosis could cause death prior to cleavage.

One potential explanation for how genetic errors contribute to pre-ZGA embryonic failure could be abnormal maternal loading of RNA and proteins during oogenesis in unisexuals compared to bisexual species. Given the large degree of sequence divergence between alleles, mis-regulation of gene expression could change the levels of maternally loaded transcripts from what is normally required to initiate early development after insemination. Improper levels could cause failure of development prior to ZGA. Future measurements of gene expression from unfertilized eggs of unisexuals compared to bisexual species (see [48]) will reveal whether any differences exist in the initial factors needed for development.

As an alternative to failures stemming from meiosis, our results are consistent with unsuccesful insemination as the primary source of embryo mortality in unisexual salamanders. For instance, lack of sperm due to low numbers of males in the ponds could result in high rates of un-activated eggs [26]. Our study did not attempt to estimate relative abundance of pure *A. jeffersonianum* vs. unisexuals in the ponds we examined, but the latter appear to greatly outnumber the former at most sites examined in Massachusetts ([16, 17, 24] JEK unpublished data). In our field observations where maternal lineage was known, unisexuals averaged 70% (range 38–100%) of the egg-mass sample among the 8 *A. jeffersonianum* ponds studied, suggesting a possibility that unisexuals outnumbered pure *A. jeffersonianum* females. However, pure *A. jeffersonianum* had lower rates of dead embryos; if sperm is the limiting factor for unisexual salamanders, such a problem does not appear to impact pure *A. jeffersonianum* females breeding in the same pond. Differential access to sperm between unisexuals and female *A. jeffersonianum* could be explained by mate selection. Female *A. jeffersonianum* are more closely related to male *A. jeffersonianum* than are unisexuals, which also carry a set of *A. laterale* nuclear genes. Thus, environmental cues triggering their breeding migration, microsite preferences during the breeding aggregation, and the pheromones they exude during courtship could put unisexuals at a disadvantage when competing for males against *A. jeffersonianum* females [27, 43, 64].

Alternatively, insemination could be impeded by locally-evolved incompatibility between host sperm and unisexuals. There may be an evolutionary incentive for males to adapt mechanisms to kill off any unisexual eggs which their sperm encounter. The resulting offspring would likely not incorporate paternal DNA while competing against offspring more genetically-related to the father. On the other hand, any genes that were incorporated into this mostly-clonal system would be subject to less generational loss, and so perhaps the long-run benefit is equal. Whether any pre-zygotic isolating mechanisms have evolved is an interesting question, particularly in light of the presence of a few populations in southeast Massachusetts completely lacking in unisexuals. Other than those populations, all *A. jeffersonianum* and *A. laterale* populations that have been examined in the state, including all sites used in this present study, also have unisexual salamanders present ([24]; JEK unpublished data).

Although we did not see different mortality rates between unisexual salamanders and *A. jeffersonianum* in the laboratory, we did see differences in the field. This discrepancy between field and laboratory results supports our conclusion that mortality among unisexual salamanders arises very early in development. In interpreting these results, it is important to recognize that the laboratory-reared embryos in our study do not represent random samples of the populations, as we specifically selected egg masses in the field that had not already begun to display mortality. Thus, for our laboratory study, we likely under-sampled egg masses with early-stage death. This is likely why the overall rate of dead embryos appears lower for unisexuals in the laboratory than in the field – because the laboratory study was focused on the portion of the population that survived past early embryogenesis.

Taken in isolation, we can infer from our laboratory results that egg masses that do not display elevated embryo mortality early in embryogenesis also do not display elevated mortality later in development. This leaves open the possibility that egg masses with elevated mortality during early embryogenesis did have a substantial number of embryos that died later in development. However, our field observations capturing representative samples from across the state support the finding that death at the early stages of embryogenesis is the primary driver of embryo mortality at the population level. In both the unisexual masses that were genetically identified and the masses of unknown maternal origin, we observed substantial percentages of dead embryos in early development, on par with the overall percent of dead eggs observed across all stages.

These field observations only count eggs as dead when the decay had advanced to the characteristic white appearance, which, as seen in our laboratory results, is substantially delayed from the time when development stops (Fig. 3). Thus, embryos that are already visibly decayed in any of these early stages likely died at or before the earliest stage of development. When analyzing field images, we observed that visibly dead embryos typically appeared as simple spheres indicating that they died in the early stages of embryogenesis, even in the egg masses with very developed embryos. However, many images were excluded from this analysis because they contained a mix of two apparently intact developmental stages: some embryos in mid- to late- stage development, and other embryos that were still in early development, but did not yet show outward signs of decay. We would not have counted such embryos as dead based strictly on the protocols in this study. While the mix of developmental stages might have indicated that there were multiple egg masses in the image, it is also very likely that they were not from a different mass, but, rather, delayed and therefore dying embryos. Development of viable embryos within an egg mass is normally synchronous [13, 42], and our observations of egg masses in the lab support the interpretation that delayed development generally leads to an eventual decay of the embryo. Thus, the true number of dead eggs at early stages of development is likely even greater than what we estimated.

In regional field surveys, the percent dead eggs observed in the earliest stages of development were actually slightly higher than the overall mean across all stages of development. This is a counter-intuitive result, as we would expect the number of dead embryos to only increase as additional ones die in each successive stage and dead embryos accumulate. It may be that embryos that died early in development decayed so much that they were unrecognizable, and thus not counted in the later stages. There may also be some other structural bias in the timing of our field surveys with respect to species distributions in the field, or recognizability of egg masses in the field. Although these field surveys are observational and therefore subject to many such biases, they offer validation for the laboratory findings that unisexual embryo mortality is not primarily driven by massive die-offs in mid or late stage development.

As we continue to pinpoint the causes of embryo mortality among unisexual salamanders, a full understanding of the processes holds the potential to inform management of these species of conservation concern, while offering insights into the evolution of sex, gene regulation, and development.

## Conclusions

We found that the phenomenon of high rates of mortality among unisexual embryos within our study region is restricted to the earliest stages of embryogenesis. Once embryos entered cleavage, we saw no difference in survival between unisexual and Jefferson salamander embryos through hatching. This suggests that embryo mortality stems from errors during oogenesis or activation. Our findings raise question about how these polyploid hybrid embryos successfully regulate development, and open the door to more studies in this fascinating system.

## Methods

### Field observations – maternal lineage known

**Fig. 6:**
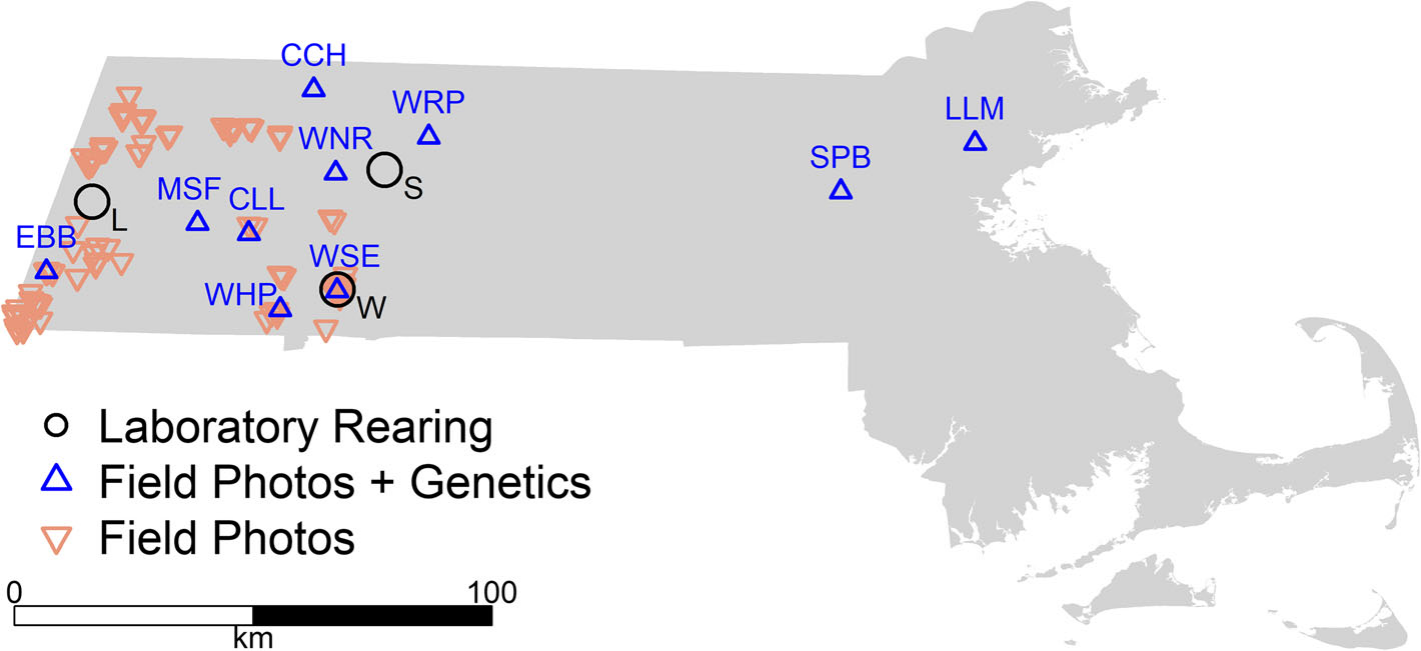
Map of field sites, with site codes identifying ponds with in-depth genetic surveys. Laboratory-reared specimens were obtained from the towns of Lenox (L), Sunderland (S), and West Springfield (W), Massachusetts, USA

We photographed and collected genetic data from 94 distinct egg masses in the unisexual salamander complex distributed among 10 ponds across Massachusetts during spring 2017 (Fig. 6), with a total of 5–11 egg masses sampled from each pond. A single observer (J.E. Kubel) conducted all surveys, photographing each egg mass in situ from multiple angles and then excising an embryo for genetic analysis. Through haphazard sampling, the goal for egg mass selection in this portion of the study was to obtain representative samples of the populations. The observer attempted to maximize independence of samples (i.e., egg masses deposited by different salamanders) to the extent that conditions (i.e., pond size and egg-mass distribution) allowed. For example, upon sampling an egg mass, he typically exited the pond and re-entered at a different location, or moved to another area within the pond at least 5 m away, before resuming sampling. To determine maternal lineage for each egg mass, we sequenced the mitochondrial D-Loop of the embryo that had been collected from the mass. We also attempted to determine a nuclear genotype (e.g., LJ, LJJ, LJJJ [44];) for each unisexual embryo sampled. For full laboratory techniques, see the supplemental methods.

For our analysis, we examined the photographs of each egg mass to identify the developmental stage(s) [58] of viable embryos. We then grouped egg masses into four stage categories: early (stages 1–19, blastula, gastrula, and the beginning of the neurula); middle (stages 20–25, end neurula and the beginning of tail bud); late (stages 26–34, gill bulge formation, nervous system lengthening); and very late (stages 35–40, gill, fin, and full larva), representing our ability to discern distinct embryo morphologies from field photographs.

We counted the number of dead embryos per mass, as evidenced by white clouds or other clumps of deteriorated embryonic matter contained within the embryonic membranes. Embryos also frequently display arrested development relative to advanced development of other embryos in the mass (i.e., other embryos having advanced to a later stage category). These likely represent embryos that have died but not yet decayed, because development of viable embryos within an egg mass is normally synchronous [13, 42]. However, we did not include these in our counts of dead embryos, because such determinations are often hard to make in a field setting due to the potential overlap of multiple egg clutches and the low-quality viewing environment.

We calculated the percentage of total dead embryos in each mass, then analyzed patterns of mortality by calculating mean percent dead embryos by stage category and by maternal lineage (i.e., *A. jeffersonianum* vs. unisexual). To test for differences in percent dead embryos among stage categories and species, we conducted mixed-effect ANOVAs, with site as a random factor, using the nlme package in R.

### Field observations – maternal lineage unknown

We also examined embryo mortality in field photographs of egg masses from 137 ponds surveyed across western Massachusetts (Fig. 6) in 2013. The surveys had previously been conducted during a separate study investigating the state distribution of *A. jeffersonianum* (Massachusetts Division of Fisheries and Wildlife – Natural Heritage and Endangered Species Program, unpublished data). Egg masses were evaluated critically both in the field (by J.E. Kubel and/or another experienced biologist) and by each photo (J.E. Kubel) during that study to assure correct identification to the *A. jeffersonianum* complex, based on morphological characteristics such as width of the interstitial space between embryonic membranes and firmness of the surrounding gelatinous matrix [12]. The only other mass-depositing ambystomatid salamander in the region is *A. maculatum*, whose egg masses are easily differentiated from those of *A. jeffersonianum* and its unisexual associates on those bases.

Our analysis of the photographic data followed that described in the previous section, except that genetic data were not available, and so we did not compare embryonic mortality between *A. jeffersonianum* and unisexuals. Furthermore, photographs from the 2013 surveys often showed multiple egg masses simultaneously. Therefore, in this data set, we pooled all embryos within an image that were sufficiently visible to characterize. We used only those photographs in which all apparently viable embryos were of the same stage category, except in the case of 9 photos where the egg masses could clearly be divided into distinct sections of two different categories. After excluding 109 photographs that were of low quality, we analyzed 663 distinct embryo sets, with a total of approximately 25,000 individual embryos.

### Laboratory-reared embryos

For laboratory rearing, we collected egg masses from local populations shortly after deposition. Our goal in this portion of the study was to track embryo mortality through the stages of development after cleavage. To ensure that we captured the early stages of development, we visited ponds soon after rains in the region triggered breeding migrations. We then selected egg masses that appeared to be freshly deposited and that had not yet begun to display visible embryo mortality.

We obtained 11 egg masses from 3 populations in developmental stages ranging from 1 to 8 ([58]; Table 1). We collected two egg masses from Sunderland on March 11, 2016, five egg masses from West Springfield on March 12, and four egg masses from Lenox on March 15 (Fig. 6). The total number of individual eggs collected was 356, with egg masses containing between 13 and 75 eggs each (mean = 32), and after discounting embryos killed for further analyses, the final sample sizes for each mass ranged from 12 to 69 (mean = 29).

We transferred egg masses to 5.5-gal glass tanks, with each tank containing eggs from a single population. Tanks were housed in a basement level space in Northfield, Massachusetts, and water levels were maintained by adding water collected from the source ponds. Water temperatures in the tanks over the duration of the study ranged from 11 °C to 16 °C. We aerated water and maintained tanks with separate equipment to minimize potential for disease transfer between source populations. We maintained natural light cycles using 6500 K full spectrum fluorescent lights on natural daylight timers.

From each egg mass, we extracted a single egg for genotyping, and transferred the embryos to ethanol. We then sequenced the D-loop portion of the mitochondria to assign maternal species lineage as either *A. jeffersonianum* or unisexual salamander. We assumed that every egg within a given mass came from the same individual salamander and, therefore, had the same maternal species lineage. We tracked embryo development with photographs of the side, bottom, and top of egg masses, as well as close up photographs of selected embryos. Upon hatching, larvae were released back in the appropriate source ponds.

To detect DNA fragmentation associated with apoptotic death of individual cells in developing embryos, we performed terminal deoxynucleotidyl transferase (TdT) deoxyuridine triphosphate (dUTP) nick end labeling (TUNEL) assays [33, 34, 38]. A modification of the procedure described in Fayzullina and Martin [33] was used. TUNEL sample specimens included 15 unisexual embryos and seven *A. jeffersonianum* embryos at various stages of development (stages 8+ through 25) taken from the lab-reared egg masses. We selected embryos that still appeared to be alive and actively developing, aiming to evenly distribute our sampling across egg masses and developmental stages. Because some egg masses had very few overall eggs, we sampled fewer eggs from these masses so as not to compromise other portions of our experiment. The number of eggs sampled per mass varied between 1 and 4 (Table 2). *Ambystoma* egg masses are surrounded by thick gelatinous membranes that hold all the eggs together within each mass. To minimize disturbance to other developing embryos when extracting embryos for TUNEL staining, we selected embryos towards the periphery of the egg masses. The samples were stored in 4% paraformaldehyde in Phosphate Buffered Saline (PBS). The samples were embedded in Fisher Healthcare™ Tissue-Plus O.C.T. Compound (OCT), and frozen on a block of dry ice. They were stored at − 80 °C until sectioning and TUNEL staining (Supplemental Methods).

**Table 2:** Stages of embryos sampled for TUNEL assays and microscopy

Lineage	Town	Mass ID	Stage
Jefferson	Sunderland	B	25
Jefferson	Sunderland	B	8+
Jefferson	West Springfield	G	9
Jefferson	West Springfield	G	9
Jefferson	West Springfield	G	23
Jefferson	Lenox	H	10
Jefferson	Lenox	H	25
Unisexual	Sunderland	A	8+
Unisexual	Sunderland	A	11
Unisexual	Sunderland	A	16
Unisexual	Sunderland	A	22
Unisexual	West Springfield	C	14
Unisexual	West Springfield	C	26
Unisexual	West Springfield	D	14
Unisexual	West Springfield	D	22
Unisexual	West Springfield	D	8+
Unisexual	West Springfield	E	25
Unisexual	Lenox	I	10
Unisexual	Lenox	I	25
Unisexual	Lenox	J	25
Unisexual	Lenox	K	10
Unisexual	Lenox	K	24

## Supplemental methods

### Genetic techniques

#### DNA preparation

Prior to using a DNeasy Blood and Tissue kit (QIAGEN, Germany), we lacerated samples into smaller pieces with a sterile razor blade, placed them into lysis solution along with proteinase K, and digested them overnight at 56 °C in a shaker incubator. We inactivated digested reactions with buffer, treated them with 4 μl (100 mg/ml) RNase A, and purified and ethanol-washed them using silica column purification. We eluted DNA from the column two times using 100 μl hot elution buffer. We then assessed DNA quality and concentration by gel electrophoresis and Qubit™ fluorometry (Life Technologies, Carlsbad, California).

#### Specific PCR conditions

For the lab-reared egg samples, we amplified the D-loop from 1 μl of extracted egg DNA using Phusion DNA polymerase (Thermo Scientific) and 2 μl of mixed 10 μM DL1/007 primer pair [59] in 25 μl total reaction volumes. We cycled each reaction on a Perkin Elmer 9600 thermocycler as follows: initial denaturation at 98 °C for 120 s, followed by 35 cycles of 98 °C:10 s; 55 °C:15 s; and 72 °C:30 s. We confirmed clean amplification by agarose gel electrophoresis and purified the PCR products by Promega (Madison, Wisconsin) Wizard SV Gel and PCR Cleanup System according to manufacturer’s protocol. We submitted samples to the University of Massachusetts Amherst Genomics and Bioinformatics Facility for sequencing from the DL1 primer.

For the field samples, we performed each reaction in 10 μl volumes using the following protocol: 2 μl GoTaq 5X Buffer; 0.8 μl 1 mM dNTP Mix; 0.4 μl of mixed 10 μM primers THR and 651 (Shaffer and McKnight 1996); 0.08 μl GoTaq® Polymerase (Promega, Madison, Wisconsin); 5.72 μl water, and 1 μl of DNA. We cycled each reaction on a BioRad T100 thermocycler (BioRad, Hercules, California) as follows: initial denaturation at 94 °C for 120 s, followed by 24 cycles of 94 °C:60 s; 48 °C:60 s; and 72 °C:60 s. We used a ramp transition rate of 0.5 °C/s for the first five cycles and did not ramp subsequent cycles. We held reactions for a final elongation step at 72 °C for 600 s. To confirm amplification, we loaded PCR reactions into a 1% agarose gel, ran them at 100 V for 25 min in 1X TAE buffer, stained them with SYBR® Safe (Life Technologies, Carlsbad, California), and visualized them under UV fluorescence. We removed unincorporated dNTPs and primers using ExoSapIT (Affymetrix, Santa Clara, California). We conducted single-strand terminator sequencing reactions in 5 μl volumes using: 1 μl GoTaq 5X Buffer; 0.5 μl BigDye v3.1 (Life Technologies, Carlsbad, California); 0.25 μl THR or 651 10 μM primer; 2.25 μl water; and 1.0 μl clean D-loop PCR product. We cycled reactions on a BioRad T100 thermocycler at 96 °C:120 s, followed by 30 cycles of 96 °C:10 s; 50 °C:0:05 s, and 60 °C:240 s. We cleaned sequencing reactions with Sephadex size exclusion media (GE Healthcare Life Sciences, Pittsburgh, Pennsylvania), suspended them in 15 μl HiDi formamide, and electrophoresed them on an ABI 3130 (Life Technologies, Carlsbad, California) capillary sequencer to generate sequence reads.

#### Microsatellite amplification

We generated two 10X primer mixes for microsatellite experiments. Mix 1 contained AjeD94, AjeD75, AjeD346, and AjeD422, and Mix 2 contained AjeD283 and AjeD378. Each primer was 2 μM concentration. We performed each multiplex PCR reaction in 10 μl volumes using 5 μl QIAGEN multiplex solution (QIAGEN, Germany); 1 μl Q-solution; 1 μl 10X primer Mix; 1 μl water, and 2 μl of DNA. We cycled each reaction on a BioRad T100 thermocycler using the following conditions: initial denaturation at 95 °C for 900 s, followed by 35 cycles of 94 °C:30 s; 57 °C (Mix1) or 58 °C (Mix2):90 s; and 72 °C:60 s, then a final elongation step at 72 °C for 1800 s. We diluted each microsatellite reaction five-fold and combined 1 μl diluted PCR product with 10.65 μl HiDi formamide and 0.35 μl GeneScan 500-LIZ™ size standard (Life Technologies, Carlsbad, California). We ran each reaction on an ABI 3500 automated sequencer to generate .fsa files for subsequent analysis.

### TUNEL assays

Samples embedded in OCT were removed from − 80 °C and allowed to equilibrate to − 20 °C in a cryostat for at least 1 h, then mounted on the cryostat chuck with OCT. 8 μm sections were cut, and each was mounted onto the charged side of a Superfrost slide, one section per slide. The sections were designated as follows: negative control, positive control, and TUNEL. The slides with sections were dried over 1–2 days in an empty pipette box slightly ajar.

The Trevigen® TACS® In situ Apoptosis Detection Kit was used. Staining and fluorescent labeling of mounted specimens were done per kit instructions, with the following modifications. In order to reduce background autofluorescence, the tissue sections were immersed in 0.1% Sudan Black B (SBB) and 70% ethanol for 20 min at room temperature. In order to remove the excess SBB, the slides were then washed three times for 5 min each in a PBS solution with 0.02% TWEEN 20.

The sections were then stained with 50 μl of Hoechst (1:2000 dilution of 10 mg/ml Hoechst stock solution in 1x PBS) for 5 min. The slides were washed three times in PBS for 1 min each, then covered with a cover slip with about 40–50 μl of ProLong Diamond Antifade Mountant (Life Technologies), wicking away excess.

Fluorescence and bright-field images were taken on a Nikon Eclipse TE2000-U Inverted Epifluorescence Microscope (Tokyo, Japan) running NIS Elements Imaging Software Version 4.30.02, equipped with an X-Cite fluorescence lamp (Lumen Dynamics) Photometrics CoolSNAP HQ camera (Roper Scientific), and Plan Fluor Ph1 DL 10X (NA 0.30) objective. Excitation and emission filters (Chroma, Rockingham, VT) in separate LEP motorized filter wheels were controlled by a MAC5000 controller (Ludl, Hawthorne, NY). LUTs adjustments were made in NIS-Elements to enhance signal and reduce background fluorescence. ImageJ plugin Scientifig was used to create the figure.

## Supplemental results

Unisexual egg mass F suffered complete embryo mortality (see animation supplement archived on Dryad, 10.5061/dryad.rxwdbrv40). None of the 20 eggs in mass F entered the first stage of division. The membranes surrounding the eggs remained irregular and lacked rigidity, unlike the firmly spherical appearance of egg membranes in other masses.

In unisexual egg mass E, the only mortality among the 24 embryos were six embryos all clustered together on the end nearest mass F, which was housed in the same tank. None of these six embryos progressed beyond stage 9. In some of these embryos, beginning in stage 2, we noticed fine mold-like filaments on the outside of the egg membranes, but we are unable to identify the mold species involved [2, 30]. In four of these embryos from mass E, we observed blebbing and cratering during early development (Stages 2–5). It is possible that a pathogen transferred from mass F to mass E; however, the embryos in mass E displayed abnormal development long before mass F displayed any mold or other signs of decay – so what the pathogen would be is not entirely clear. Conversely, the abnormal membrane morphology suggests that mass F was apparently already failing to develop before we collected it, and mortality was likely not related to pathogens transferred from mass E.

In unisexual egg mass A, in six of the 35 embryos, we observed a ballooning structure emerging from the abdomen in mid-development stages (stages 23–25), which continued developing until later stages (stages 26–33) prior to death. Two other embryos in mass A aborted development at stage 9 and were engulfed by mold when neighboring embryos were in stages 25 and 26.

In *A. jeffersonianum* egg mass B, 11 of the embryos aborted development at stages 21 through 23, and were consumed by mold when the other embryos in the mass were in very late development (stages 35–40). Another embryo from mass B showed a streak of mold inside the egg membrane in stage 4, and failed to progress past stage 8. One other embryo in mass B caved inward at stage 9.

In unisexual egg mass K, two of 22 embryos were engulfed by blooms of iron bacteria associated with the brackets anchoring the masses. Blooms of these bacteria were observed in the source pond. In mass K, there was also abnormal development in two embryos that died in stages 10 and 24, and three other embryos that died in stages 12, 14, and 35.

Masses H and I were also surrounded by iron bacteria blooms. In Jefferson egg mass H, 10 of the 24 embryos died in stages 18 through 38. In unisexual mass I, one of the 18 embryos died at stage 12 and one died between stage 35 and 39. The membranes of two other embryos in mass I were entirely engulfed in the iron bacteria, but the larvae hatched nonetheless.

In unisexual mass C, one of 15 embryos was engulfed by mold at stage 23, and one other embryo showed signs of mold in the membrane in stage 30, but the larva advanced to stage 40 and then remained within the membrane alive for longer than any other larva.

In unisexual mass D, four of 23 embryos aborted development at stages 9, 12, 18, and 39.

Out of 59 embryos in *A. jeffersonianum* mass G, one embryo was engulfed in white mold when neighboring embryos were at stage 31, but it was obscured by the surrounding embryos, so we did not observe prior stages of development in this embryo.

## Data Availability

Associated data tables, images, and R Scripts are archived in Dryad: 10.5061/dryad.rxwdbrv40. Associated genetic sequences are archived at NCBI GenBank under accession numbers MK185107 - MK185211.

## Abbreviations

B: Blastula staged
dUTP: deoxyuridine triphosphate
H: Heartbeat
MBT: Mid-blastula transition
MZT: Maternal-to-zygotic transition
N: Neurulation
OCT: Fisher Healthcare™
PBS: Phosphate buffered saline
SBB: Sudan black B
T: Tail-bud
TdT: Terminal deoxynucleotidyl transferase
Tissue-Plus: O.C.T. Compound
TUNEL: TdT dUTP nick end labeling
UTP: Deoxyuridine triphosphate
ZGA: Zygotic genome activation
